# Prevalence of *Coxiella burnetii* in unpasteurized dairy products using nested PCR assay

**Published:** 2018-08

**Authors:** Fargol Abdali, Saeid Hosseinzadeh, Enayat Berizi, Siamak Shams

**Affiliations:** 1 Nutrition Research Center, Department of Food Hygiene and Quality Control, School of Nutrition and Food Sciences, Shiraz University of Medical Sciences, Shiraz, Iran; 2 Department of Food Hygiene and Public Health, School of Veterinary Medicine, Shiraz University, Shiraz, Iran; 3 Department of Environmental Health Ward, Shiraz University of Medical Sciences, Shiraz, Iran

**Keywords:** Q fever, *Coxiella burnetii*, Nested-PCR, Phylogenic analysis

## Abstract

**Background and Objectives:**

Q fever is a worldwide disease which is common between humans and livestock. This disease is created by an obligate intracellular Rickettsia called *Coxiella burnetii (C. burnetii).* The aim of this study was to determine the prevalence of *C. burnetii* in unpasteurized dairy products in Shiraz.

**Materials and Methods:**

In this study (from summer 2016 to winter 2016), 238 non-pasteurized dairy products, (48 raw milk, 48 yogurt, 46 cheese, 48 dough and 48 ice cream samples) were collected from the retail market and analyzed using a nested PCR assay.

**Results:**

This study showed that 20 samples (8.4%), out of the 238 unpasteurized dairy products, were positive for *C. burnetii* as follows: 13 out of 48 (27.08%) raw milk, 3 out of 48 (6.25%) yogurt, 2 out of 46 (4.35%) cheese, 2 out of 48 (4.16%) dough, and 0 out of 48 ice cream samples.

**Conclusion:**

The present study suggests that unpasteurized dairy products are the main sources of *C. burnetii* in Shiraz, Southern Iran; thus, the consumption of pasteurized milk and dairy products is a valuable method to prevent the disease in humans.

## INTRODUCTION

*Coxiella burnetii* is a zoonotic pathogen. The disease caused by this organism in humans and livestock is called Q fever and coxiellosis, respectively. This agent is a compulsory intracellular parasite that propagates into monocytes and macrophages (1). A range of animals, such as cows, sheep, goats, dogs, cats, reptiles, amphibians, birds (domesticated and wild), fish and a number of ticks can be contaminated by this organism (2). Cattle, sheep and goats are the main sources of disease to humans (1). Contaminated animals shed through the stool, urine, uterine secretions and milk to the environment. Transmission of the organism to humans could happen via three major routes; through the respiratory tract (aerosols), digestive system (consumption of contaminated products) and skin (bite of ticks and dermal ulcers) (3). The most common means of infection in humans is through inhalation of contaminated aerosols or consumption of milk and unpasteurized dairy products (4). Today, milk and its products are one of the major trading activities; to the extent that even in some countries, the per capita consumption of milk in the community is mentioned as an indicator of progress and development. Milk and dairy products play important roles in human nutrition because of their high nutritional value. On the other hand, due to the presence of most of the elements and food constituents in them, they are potentially a good environment for the growth and activity of many pathogenic micro-organisms (5).

Humans are very sensitive to the disease, and only a small number of bacteria can cause a serious infection in them. Clinical symptoms of the acute Q fever include a sudden headache, fever, pneumonia, fatigue, chills, headache, muscle aches, sweating, coughing, nausea, vomiting, chest pain, diarrhea, skin rash, myocarditis, pericarditis, meningoencephalitis and even death. In chronic forms of the infection, endocarditis, bone and joint involvement, vascular infections, chronic lung infection and chronic fatigue syndrome, have repeatedly been reported (2, 3). In addition, the infection can lead to abortion and stillbirth in pregnant women and also classified as the B group of the most common carcinogens (2, 6).

Q fever is widely spread throughout the world. In recent years, there have been reports of the infection in Belgium, Netherlands, United States, Canada, Saudi Arabia, Bahrain, Qatar, the United Arab Emirates, Egypt, Oman and Japan (1). Precise information on the prevalence of this infection in Iran is not available. The first report of Q fever in Iran was recorded in 1952. In this study, the presence of the microorganism was confirmed in two patients in Abadan (Southwest of Iran) using serological assay. The evidence of infection was also recorded in Sabzevar (Northeast of Iran) in 46.5% of the samples (7). In 2011, Khalili et al. reported the prevalence of this infection in ruminant and febrile patients by conducting early serological studies in Southeastern Iran (8). Moreover, 14% of the febrile patients showed sero-positive results of Q fever in Iran (9). A meta-analysis performed in 2016 revealed prevalence of 19.8% and 32.86% of IgG phases I and II of *C. burnetii* in the patients (10).

It must be noted that *C. burnetii* is a class of bacterium that could be recognized by classical methods such as cultivation which is confronted with limitations and serological and, recently, molecular based techniques (3). Q fever is essentially an occupational illness; however, the infection is widely transmitted to humans through inhalation of contaminated aerosols and/or consumption of unpasteurized dairy products which may cause the illness among the general population (11).

Considering the important consequences of the infection and the limited available information on its prevalence in southern Iran, this study was conducted to determine contamination rates of *C. burnetti* in milk and unpasteurized dairy products in Shiraz using a nested PCR assay.

## MATERIALS AND METHODS

### Sample collection.

In this cross-sectional descriptive study (from summer 2016 to winter 2016), 238 unpasteurized dairy products samples, (48 raw milk, 48 yoghurt, 46 cheeses, 48 dough and 48 ice cream samples) were randomly collected from the retail market in Shiraz and transported to the laboratory in sterile containers on ice which was later stored at −20°C until further use.

### DNA Extraction.

DNA from samples were extracted using a DNA extraction kit (Bioneer, South Korea) as per the manufacturer’s instructions. Two hundred milligrams of each sample was used. The quantity of DNA was recorded using ANG 100 spectrophotometer (Nanodrop Technologies, USA).

### Nested PCR Assay.

PCR was performed on the DNA extracted from all the samples (n=238); two pairs of species-specific primers (Table 1) were used to amplify a region (438 bp) of the 27 kDa outer membrane protein (*com1*) gene (12). Each 25 μL reaction consisted of 12.5 μL master mix, 1 μM of each primer, 2 μL of DNA and 8.5 μL of PCR grade water. Amplification was performed in a Thermal Cycler-LifePro (Bioer Technology, China) using the following cycling conditions: denaturation at 94°C for 3 min and then 35 cycles of 94°C for 45 sec, 56°C for 45 sec, and 72°C for 45 sec, followed by a final extension at 72°C for 5 min. The second run of PCR was conducted using OMP-3 and OMP-4 primers. Two microliter of the PCR product was employed as a template DNA. Other conditions (time, temperature, reaction cycles and the volume for each reaction) were the same as the initial reaction. Furthermore, a positive control (Nine Mile strain, ATCC VR-615) and a negative control (Ultra Pure Water DNase and RNase-free, Cinnagen, Tehran, Iran) were incorporated in each assay. For each sample, a volume of 8 μL of PCR product was run on a 1.5% agarose gel in Tris-acetate-EDTA (TAE) running buffer stained with safe mode DNA stain (SinaClon, Tehran, Iran) and visualized by a UV transilluminator.

**Table 1.** Primers used in the present study

	**Primer**	**5′3′ →**	**Amplicon size (bp)**	**Ref.**
1^st^ PCR	Cox1	AGTAGAAGCATCCCAAGCATTG	501	(12)
	Cox2	TGCCTGCTAGCTGTAACGATTG		
Nested PCR	Cox3	GAAGCGCAACAAGAAGAACAC	438	
	Cox4	TTGGAAGTTATCACGCAGTTG		

### DNA sequencing and data analysis.

The sequencing of 16s rRNA PCR product was performed on the amplified genes using the QIAquick gel extraction kit (Bioneer, USA), as described by the manufacturer. The pure products were subjected to sequencing (Macrogen, South Korea). After sequencing, they were analyzed using Mega7 software.

### Statistical analysis.

All data were analyzed by SPSS (version 21; SPSS Inc., Chicago, IL, USA). Logistic regression analysis (OR ratio) was used to evaluate the proportion of contamination during winter and summer among various products. The level of statistical significance was set at P<0.05.

## RESULTS

**Fig. 1.**
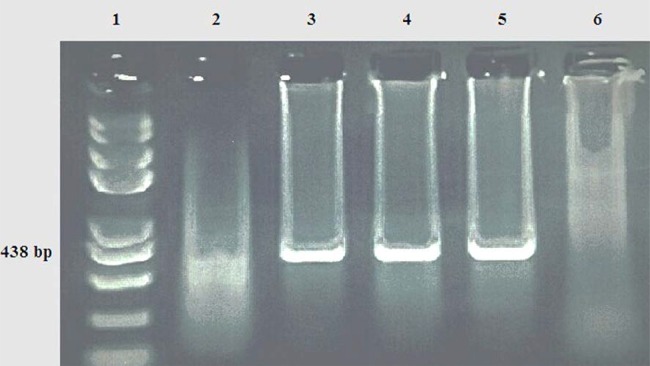
Gel electrophoresis of products of nested PCR on DNA template of some isolates. Lines 1: 100 kb DNA ladder, Lanes 2: negative control, lane 3: positive control (pure DNA of the bacterium), lanes 4 & 5: positive samples and Lane 6: negative samples (no template).

**Fig. 2.**
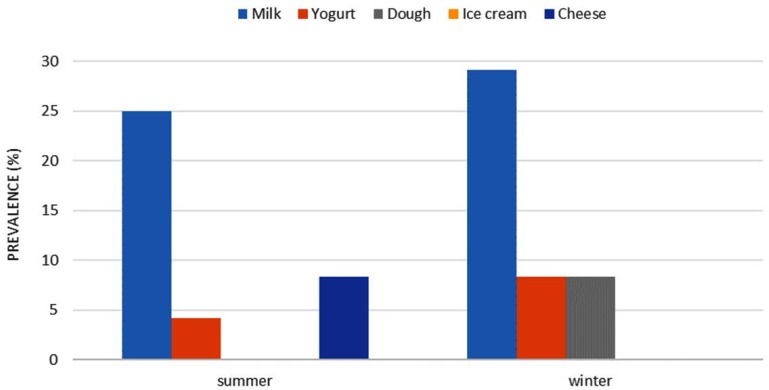
Frequency of *C. burnetii* in non-pasteurized dairy product samples in summer and winter.

In this study, 238 unpasteurized dairy products were tested for *C. burnetii* using a nested PCR assay. In total, 20 samples (8.4%) including 13 out of 48 raw milk (27.08%), 3 out of 48 yogurt (6.25%), 2 out of 46 cheese (4.35%), 2 out of 48 dough (4.16%) and 0 out of 48 ice cream were reported positive (Table 2). All the positive samples were 100% identical to each other at the *com 1* gene. This was confirmed by the observation of a 438 bp fragment in the PCR assay (Fig. 1). Also, statistical analysis (OR) showed that the probability of contamination in yogurt, dough and cheese were lower (82%, 88.3%, 87.8%, respectively) compared to the raw milk (P <0.05) (Table 2). In winter, higher numerical rates of contaminations were observed in all dairy products except for the cheese (P> 0.05) (Fig. 2); however, the overall contaminations of the products were 22% higher in the summer time (OR: 1.22, P> 0.05).

**Table 2.** Frequency of *C. burnetii* in unpasteurized dairy product samples

**Product type**	**Total number**	**Positive number**	**Positive number in winter**	**Positive number in summer**	**Total Prevalence Odds ratio**	**(OR)**	**P value**
Milk	48	13	7	6	27.08 (95% CI:15.1–42.9)	-	-
Yogurt	48	3	2	1	6.25 (95% CI:6–14.8)	0.179	0.011
Dough	48	2	2	0	4.16 (95% CI:0–10.1)	0.117	0.007
Cheese	46	2	0	2	4.35 (95% CI:0–10)	0.122	0.008
Ice cream	48	0	0	0	0	0.000	0.997
Total	238	20	11	9	8.4 (95% CI:5–12.2)	-	-

**Fig. 3.**
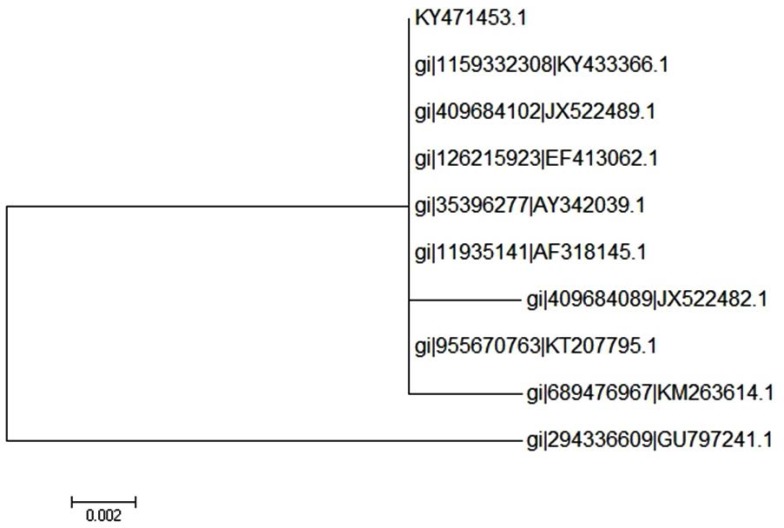
Phylogenetic tree showing the relationship between the 16S rRNA gene sequences from representative isolates of *Coxiella* from raw milk. Eliminate the branches with bootstrap less than 50%

BLASTn used for the comparison of the sequences of the samples, against the nucleotide database, did return a significant result to *C. burnetii*. The sequences of samples were submitted at NCBI with the accession number KY471453 for the *com 1* gene. The result of the phylogenetic analysis of the isolates is shown in Fig. 3. Our analysis represented the most homology to one of the previously reported Iranian isolates and was similar to those reported elsewhere (100%).

## DISCUSSION

Although Q fever is a primary occupational disease, milk and its contaminated products can play important roles in the epidemiology of the infection (13). A study in the United States showed the occurrences of 10.7% and 0.7% of the infection between different populations. Similar results have also been reported from England, Bulgaria, Slovakia and Spain (11, 14). According to a study conducted in Sweden in 2007, of 359 cow milk samples collected from cheese factories, 17 samples (7.4%) were contaminated with *C. burnetii* (15). In a similar study done in Turkey, 3.5% of the 400 milk samples from 23 herds of sheep were reported positive for *C. burnetii* (16). In another study conducted in the United States in 2010, of 21 milk samples, 9 samples were reported positive for *C. burnetii* (17). Also, the prevalence of 56.6% in the cow milk samples was reported using PCR (18). Studies from various geographical areas of Iran revealed prevalence of 0–48% in milk samples. For instances, the rate of contamination in central (Chahrmahal va Bakhtiari), southern (Jahrom), northwest (Bonab) and southwest (Khuzestan) areas were respectively 6.2%, 11%, 26% and 1.1%. Moreover, presence of *C. burnetii* was confirmed in total of 9.72% of cattle, 2.54% of sheep and 2.6 % of goat bulk milk samples (19).

The most important reasons that could be cited for the difference reported in the prevalence of *C. burnetii* in dairy products in different parts of the world are the diversities in climate and environmens of the geographical areas, the type of survey, the type and number of samples taken and the season in which sampling took place (2, 14, 20, 21).

In the present study, the highest prevalence of infection was recorded in winter although there was no significant statistical difference in the prevalence of the infection in different seasons of the year. In a study carried out by Rahimi et al. in Isfahan province, using nested PCR assay from 2008 to 2009, the results showed different rates of contamination in different seasons; however, the highest incidence (8.6%) was observed in winter, while all 65 samples taken in the summer were negative (22). Fretz et al. reported a higher infections rate of cow milk in winter (12) because of high levels of excretion of the microorganisms from uterine secretion, urine, feces and milk of infected cows.

In this study, a number of yogurt, cheese, and dough samples were positive for the presence of *C. burnetii*; the presence of *C. burnetii* in these samples is probably the result of inadequate milk processing prior to production. Some studies show that high temperature and short time pasteurization (HTST) can eliminate this microorganism (11). Therefore, the risk of infection is higher with the consumption of unpasteurized dairy products.

Given that *C. burnetii* is a class of microorganism that is recognized by classical methods such as cultivation, which have some limitations, the use of diagnostic methods based on molecular biology techniques can contribute to the rapid diagnosis and management of the disease (3). Similar to other zoonotic diseases, control of Q fever in humans depends largely on controlling infection in animals. Legislation on compulsory removal of contaminated animals, the transport, and obligatory vaccination of livestock are also important tools to control the disease (23).
